# Necrotizing enterocolitis with gastric perforation in a 24-day old preterm neonate

**DOI:** 10.62838/jccm-2026-0023

**Published:** 2026-07-27

**Authors:** Tamas Toth, Reka Borka-Balas, Manuela Cucerea, Radu-Alexandru Prisca

**Affiliations:** Institution Organizing University Doctoral Studies (IOSUD) George Emil Palade University of Medicine, Pharmacy, Science and Technology of Targu Mures; Pediatric Surgery and Orthopedics Department, Targu Mures Emergency Clinical County Hospital, Targu Mures, Romania; Pediatric Clinic I, Targu Mures Emergency Clinical County Hospital; George Emil Palade University of Medicine, Pharmacy, Science and Technology of Targu Mures, Targu Mures, Romania; Neonatology Department, Targu Mures Emergency Clinical County Hospital; George Emil Palade University of Medicine, Pharmacy, Science and Technology of Targu Mures, Targu Mures, Romania; Pediatric Surgery and Orthopedics Department, Targu Mures Emergency Clinical County Hospital, Targu Mures, Romania

**Keywords:** neonatal gastric perforation, necrotizing enterocolitis, extremely low birth weight, prematurity, pneumoperitoneum

## Abstract

**Introduction:**

Neonatal gastric perforation (NGP) is a rare, life-threatening surgical emergency that predominantly affects premature and extremely low birth weight (ELBW) infants and remains associated with high mortality.

**Case presentation:**

A 600 g female infant born at 25/26 weeks of gestational age developed necrotizing enterocolitis (NEC) during the third postnatal week and deteriorated with abdominal distension and pneumoperitoneum. Emergency laparotomy on day 24 revealed a single posterior gastric wall perforation with circumferential necrotic margins; the nasogastric tube tip was located at the defect. After minimal debridement and primary two-layer closure, the infant survived a prolonged intensive care course complicated by recurrent sepsis, cholestasis, bronchopulmonary dysplasia, and later adhesive obstruction requiring adhesiolysis.

**Conclusions:**

Gastric perforation may represent an uncommon manifestation of severe NEC in ELBW infants. Delayed onset, necrotic margins, and systemic inflammatory deterioration may favor ischemic NEC-related injury over iatrogenic trauma. Early radiographic evaluation and prompt surgical exploration are crucial for survival.

## Introduction

Necrotizing enterocolitis (NEC) is the most frequent gastrointestinal emergency in preterm infants with an incidence of 5–10% in very low birth weight infants [1]. It is characterized by intestinal inflammation and ischemic necrosis that may progress to perforation and systemic sepsis [2]. The diagnosis is based on feeding intolerance, abdominal distension, hematochezia, laboratory evidence of inflammation, and radiologic signs such as pneumatosis intestinalis or pneumoperitoneum. Differential diagnosis includes spontaneous intestinal perforation and other neonatal gastrointestinal perforations. Management ranges from bowel rest and broad-spectrum antibiotics to surgery when perforation or clinical deterioration occurs.

NEC is a leading cause of perforation in this population, as shown in several institutional series [1,2,3,4]. Gastric involvement is distinctly rare, likely represents an extreme manifestation of systemic inflammation and hypoperfusion rather than the typical distribution of disease [2].

Neonatal gastric perforation (NGP) is a life-threatening condition with a mortality rate of 20–50% and increases in the presence of sepsis, metabolic acidosis, thrombocytopenia, or circulatory collapse [5,6,7].

It accounts for approximately 10–16% of neonatal gastrointestinal perforations and occurs predominantly in premature and extremely low birth weight (ELBW) infants [8,9].

We report an ELBW infant who developed posterior gastric perforation as a localized manifestation of NEC, with favorable gastrointestinal outcome. The aim of this paper is to highlight gastric perforation as an uncommon localized manifestation of NEC in an ELBW infant and to discuss its diagnostic and therapeutic implications.

## Case presentation

### Presenting concerns

A 600g female infant born at 25/26 weeks gestational age was delivered vaginally to a 27-year-old gravida II, para I mother with unknown antenatal history. Two doses of antenatal dexamethasone were administered prior to delivery. Meconium-stained amniotic fluid suggested fetal distress. Apgar scores were 7 at 1 minute, 8 at 5 minutes, and 8 at 10 minutes.

### Clinical findings

Postnatal examination revealed generalized hypotonia, respiratory rate 76 breaths/min, heart rate 165 beats/min, and oxygen saturation 90–92% in room air.

Immediately after birth, the neonate required noninvasive positive pressure ventilation (NIPPV) with peak inspiratory pressure/positive end-expiratory pressure (PIP/PEEP) 16/5.5 cmH_2_O, inspiratory time 0.4 s, respiratory rate 45 breaths/min, and fraction of inspired oxygen (FiO_2_) 30%.

A nasogastric tube was inserted, surfactant was administered using the Less Invasive Surfactant Administration (LISA) technique, and an umbilical venous catheter was placed. Minimal enteral feeding with maternal breast milk was initiated on day 0.

On day 9, respiratory support was transitioned to nasal continuous positive airway pressure (nCPAP) (PEEP 5.5 cmH_2_O, FiO_2_ 25%). Echocardiography showed ostium secundum atrial septal defect with left-to-right shunt and a patent ductus arteriosus. Empiric antibiotic therapy with ampicillin–sulbactam and amikacin was started for elevated inflammatory markers.

At day 14, intermittent gastric residuals were observed and minimal enteral feeding was continued.

At day 17, the infant developed tachycardia, persistent gastric residuals, vomiting, and mucus-containing stools. Clinical and laboratory findings indicated the onset of NEC, and antibiotic therapy was escalated to meropenem.

At day 21, the patient deteriorated with recurrent episodes of apnea and bradycardia, abdominal distension, and pathological gastric aspirates. Enteral feeding was stopped and synchronized intermittent positive pressure ventilation (SIPPV) was initiated (PIP/PEEP 15/5 cmH_2_O, respiratory rate 45 breaths/min, inspiratory time 0.36 s, FiO_2_ 45%). Dopamine infusion was administered for hemodynamic support, along with pentoxifylline and intravenous immunoglobulin.

### Diagnostic focus and assessment

**Fig. 1.**
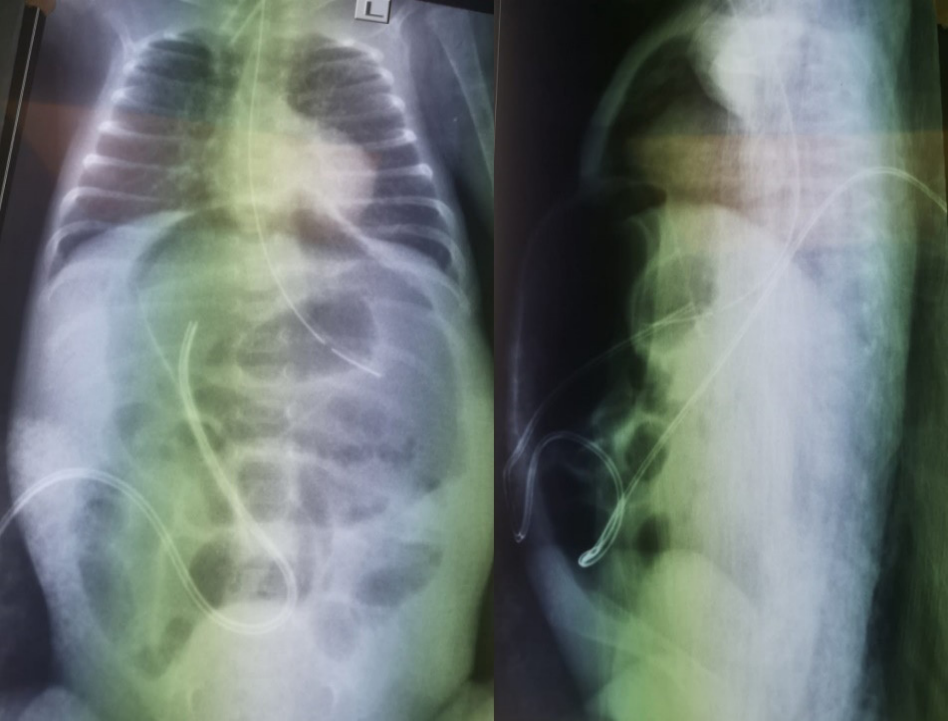
Abdominal X-ray: (A) Anterior-posterior view: thickened, dilated bowel loops marked with arrow (B) Lateral decubitus view: pneumoperitoneum – marked with arrow

At day 24, progressive abdominal distension, vomiting, constipation, and worsening clinical status prompted abdominal radiography, which demonstrated pneumoperitoneum (Figure 1). Laboratory evaluation showed markedly elevated inflammatory markers: procalcitonin 8.79 ng/mL, C-reactive protein 26.6 mg/L, white blood cell count 7.47 × 10^3^/µL, and platelet count 215 × 10^3^/µL.

The neonate was transferred to the pediatric surgery unit with a diagnosis of acute abdomen requiring immediate surgical intervention.

### Therapeutic focus and assessment

**Fig. 2.**
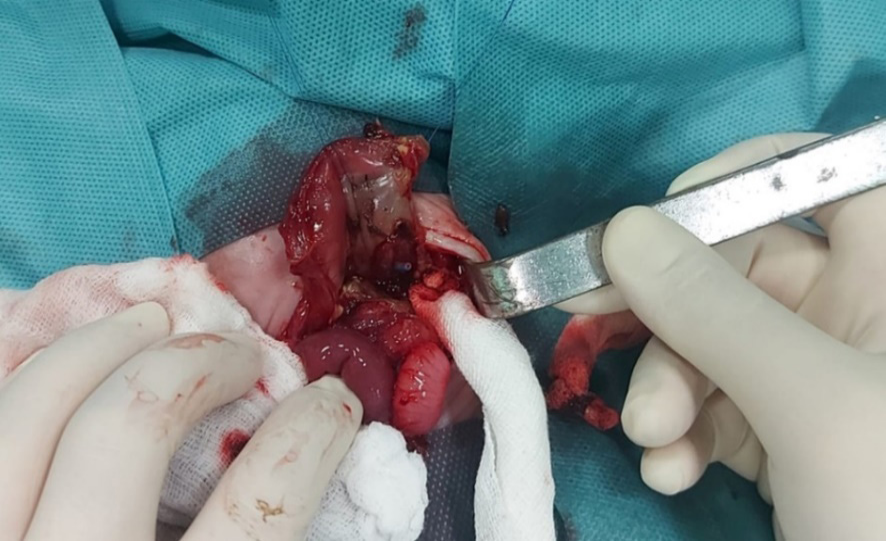
Intraoperative finding: gastric wall perforation with the presence of the nasogastric tube tip (circled)

The exploratory laparotomy revealed a single posterior gastric wall perforation measuring approximately 1 × 1 cm with circumferential necrotic-appearing margins. The nasogastric tube tip was located at the perforation site (Figure 2).

Despite this finding, the macroscopic appearance favored ischemic necrosis rather than an acute mechanical laceration. Systematic inspection of the small and large bowel showed no additional perforations or transmural necrosis. Minimal debridement of the perforation edges was performed, followed by primary two-layer closure of the gastric wall. Histopathological examination was not accomplished because no full-thickness specimen was resected.

Postoperatively, the patient remained intubated with stable oxygenation (oxygen saturation 94% on FiO_2_ 30%). Inotrope treatment was continued for three days. Enteral feeding was gradually reintroduced after five postoperative days.

### Follow-up and monitoring

By day 34, she was extubated and returned to nCPAP (PEEP 5 cmH_2_O, FiO_2_ 30%). The subsequent clinical course was complicated by multiple infectious episodes, including coagulase-negative *Staphylococcus* bacteremia and later methicillin-resistant *Staphylococcus aureus* sepsis associated with *Clostridioides difficile* enterocolitis. Cholestasis developed and was treated with ursodeoxycholic acid. Hydrocortisone therapy was introduced for the incipient bronchopulmonary dysplasia. Progressive retinopathy of prematurity required intravitreal bevacizumab administration. Cardiac therapy with propranolol and captopril was initiated.

Specific drug manufacturer details were not retrievable from the retrospective medical record. All medications were administered according to institutional neonatal intensive care protocols and contemporary standard-of-care guidelines.

At day 100, recurrent abdominal distension, vomiting, and bilious gastric aspirates recurred. The abdominal radiography showed dilated bowel loops with air–fluid levels. Repeat laparotomy revealed interileal adhesions without perforation or ischemia, adhesiolysis was performed. Enteral feeding was resumed after four days postoperatively.

After 115 days of hospitalization, the infant was discharged at 2150 g with cardiology, neurology, and physiotherapy follow-up.

At 10 months of corrected age, she weighs 5 kg and measures 62 cm. She continues propranolol and captopril therapy and receives physiotherapy and sensory integration therapy, without gastrointestinal complications at the time of last follow-up, she tolerates full oral feeding.

## Discussion

This case illustrates an uncommon presentation of NEC with isolated gastric perforation in an ELBW infant. Unlike typical NEC involving the terminal ileum or colon, the lesion was localized to the posterior gastric wall. The delayed onset in the third postnatal week, systemic inflammatory deterioration, and necrotic perforation margins supported an ischemic NEC-related mechanism rather than primary traumatic perforation.

NGP can be a life-threatening condition with potential long-term effects such as iron-deficient anemia, steatorrhea, and developmental delay, particularly in ELBW infants [8,9,10]. Early diagnosis matters because deterioration can be abrupt [11]. The initial presenting symptom is abdominal distension with emesis. The differential diagnosis includes all the neonatal intestinal obstruction diagnoses, septicemia, and NEC.

The etiology of NGP is multifactorial and incompletely understood. Possible mechanisms include hypoxic–ischemic injury, barotrauma from assisted ventilation, nasogastric tube–related trauma, drug exposure (e.g., corticosteroids, indomethacin), and functional obstruction due to gastric atony or pylorospasm [8,10,11]. Developmental weakness or focal absence of the gastric muscular layer has also been described, potentially predisposing to stomach rupture when intragastric pressure rises [12,13]. In these cases perforations often occur early—frequently within the first postnatal week—and may present even in term neonates.

In contrast, gastric perforation occurring during NEC is distinctly uncommon. NEC typically involves the terminal ileum and colon. Gastric injury can occur during episodes of severe systemic inflammation and circulatory instability, particularly in ELBW infants with immature autoregulation, anemia, and prolonged ventilatory or inotropic support [1,14,15].

In the present case, several features favored a NEC-related ischemic mechanism.

First, symptom onset and clinical deterioration occurred in the third postnatal week, matching the typical timing of NEC in very preterm infants [1].

Second, the infant presented recurrent systemic inflammatory episodes and prematurity-associated morbidities that match with severe NEC as a systemic disorder (e.g., bronchopulmonary dysplasia, retinopathy of prematurity, cholestasis, recurrent sepsis) [2,14].

Third, the perforation edges appeared circumferentially necrotic rather than sharply demarcated. The presence of the nasogastric tube tip at the defect raises the alternative possibility of iatrogenic perforation. Upper gastrointestinal perforations related to tube placement have been described in fragile preterm infants, with higher risk during difficult insertions or when performed by inexperienced staff [16]. However, tube-related injuries typically present soon after insertion and may show sharp laceration margins.

In our patient, the tube had been in place since the first days of life, and the perforation developed nearly three weeks later in the context of NEC and systemic deterioration, making primary traumatic perforation less likely.

Operative management depends on the number of perforations, tissue viability, and physiologic stability. For isolated defects with viable surrounding tissue, limited debridement and primary two-layer closure are widely recommended [9,10]. In cases with diffuse necrosis or multiple perforations, more extensive approaches such as partial gastrectomy or longitudinal sleeve gastrectomy have been reported, provided that vascularity of the lesser curve is preserved [17].

In the present case, the isolated posterior perforation and absence of bowel necrosis allowed primary repair, without postoperative complications and preserved gastric function.

Finally, the long and complicated postoperative trajectory illustrates that survivors often face substantial morbidity driven by prematurity and systemic inflammation rather than the gastric repair itself. ELBW infants requiring abdominal surgery frequently have prolonged hospitalization and complications such as recurrent sepsis, cholestasis, bronchopulmonary dysplasia, and later adhesive obstruction in ELBW surgical cohorts [14,18,19,20]

The favorable gastrointestinal outcome at 10 months’ corrected age in this infant adds to the limited literature suggesting that, with early recognition and prompt operative management, survival with preserved gastrointestinal function is increasingly achievable even after severe disease.

## Conclusions

Gastric perforation is an uncommon but important potential manifestation of severe NEC in ELBW infants. This case is original due to the isolated posterior gastric involvement and favorable gastrointestinal outcome despite extreme prematurity and severe systemic illness.

When a critically ill premature neonate deteriorates with abdominal distension and pneumoperitoneum, the stomach should be considered among possible sites of perforation.

Delayed onset, circumferential necrotic margins, and concomitant systemic inflammatory features may favor ischemic NEC-related injury over iatrogenic trauma.

Prompt radiographic evaluation and early surgical exploration remain key determinants of survival and long-term outcome.

This report contributes to the limited literature on NEC-associated gastric perforation and may assist neonatologists and pediatric surgeons in differentiating ischemic NEC-related injury from iatrogenic gastric trauma.

## References

[1] Yee WH, Soraisham AS, Shah VS (2012). Incidence and timing of presentation of necrotizing enterocolitis in preterm infants. Pediatrics.

[2] Neu J, Walker WA (2011). Necrotizing Enterocolitis. N Engl J Med.

[3] Gerçel G, Anadolulu AI Neonatal Gastrointestinal Perforations: A 4-year Experience in a Single Centre. Afr J Paediatr Surg.

[4] Sakellaris G, Partalis N, Dede O (2012). Gastrointestinal perforations in neonatal period: experience over 10 years. Pediatr Emerg Care.

[5] Huang Y, Lu Q, Peng N (2021). Risk factors for mortality in neonatal gastric perforation: a retrospective cohort study. Front Pediatr.

[6] Mengying C, Pengfei C, Jinfeng H, Yi W, Wei L, Zhenhua G (2024). Visualization of Risk Factors and Predictive Models for Early Death of Neonatal Gastric Perforation. Clin Pediatr (Phila).

[7] Hyginus EO, Jideoffor U, Victor M, N OA (2013). Gastrointestinal perforation in neonates: aetiology and risk factors. J Neonatal Surg.

[8] Terui K, Iwai J, Yamada S ichi (2012). Etiology of neonatal gastric perforation: a review of 20 years’ experience. Pediatr Surg Int.

[9] Lin CM, Lee HC, Kao HA (2008). Neonatal gastric perforation: report of 15 cases and review of the literature. Pediatr Neonatol.

[10] Kara CS, İlÇe Z, Celayır S, Sarimurat Nü, Erdogan E, Yeker D (2004). Neonatal gastric perforation: review of 23 years’ experience. Surg Today.

[11] Garge SS, Paliwal G (2020). Neonatal gastric perforation: our experience and important preoperative and intraoperative caveats to prognosticate and improve survival. J Indian Assoc Pediatr Surg.

[12] Yang T, Huang Y, Li J (2018). Neonatal gastric perforation: case series and literature review. World J Surg.

[13] Shaw A, Blanc WA, Santulli TV, Kaiser G (1965). Spontaneous rupture of the stomach in the newborn: a clinical and experimental study. Surgery.

[14] Peter C, Abukhris A, Brendel J, Böhne C, Bohnhorst B, Pirr S (2023). Growth and duration of inflammation determine short- and long-term outcome in very-low-birth-weight infants requiring abdominal surgery. Nutrients.

[15] Saraç M, Bakal Ü, Aydın M (2017). Neonatal gastrointestinal perforations: the 10-year experience of a reference hospital. Indian J Surg.

[16] Gander JW, Berdon WE, Cowles RA (2009). Iatrogenic esophageal perforation in children. Pediatr Surg Int.

[17] Reyna-Sepulveda F (2017). Neonatal sleeve gastrectomy for multiple gastric perforations: a case report. J Neonatal Surg.

[18] Attridge JT, Herman AC, Gurka MJ, Griffin MP, McGahren ED, Gordon PV (2006). Discharge outcomes of extremely low birth weight infants with spontaneous intestinal perforations. J Perinatol.

[19] Shah J, Singhal N, Da Silva O (2015). Intestinal perforation in very preterm neonates: risk factors and outcomes. J Perinatol.

[20] Emil S, Davis K, Ahmad I, Strauss A (2008). Factors associated with definitive peritoneal drainage for spontaneous intestinal perforation in extremely low birth weight neonates. Eur J Pediatr Surg.

